# Enhanced Soybean Immunity to the Soybean Mosaic Virus Through RNA Interference Targeting the *CP* Gene

**DOI:** 10.3390/plants15030430

**Published:** 2026-01-30

**Authors:** Tao Wang, Le Gao, Liqun Wang, Rui Ren, Rui Zhai, Xu Wang, Fuming Xiao, Long Yan, Xiaotong Lei, Tongtong Jin, Haijian Zhi

**Affiliations:** 1 National Center for Soybean Improvement, National Key Laboratory for Crop Genetics and Germplasm Enhancement, Key Laboratory of Biology and Genetic Improvement of Soybean-Ministry of Agriculture, Nanjing Agricultural University, Nanjing 210095, China; wt414210391@163.com (T.W.); gaole@bvca.edu.cn (L.G.); wanglq1124@126.com (L.W.); renruinjau@163.com (R.R.); zhai_rui_nyr@163.com (R.Z.); 2 Handan Academy of Agricultural Sciences, Handan 056001, China; 18931066359@163.com (X.W.); 13930083220@163.com (F.X.); lxt990209@163.com (X.L.); 3 Department of Horticulture, Beijing Vocational College of Agriculture, Beijing 102442, China; 4 College of Agronomy, Henan Agricultural University, Zhengzhou 450046, China; 5 Hebei Laboratory of Crop Genetics and Breeding, National Soybean Improvement Center Shijiazhuang Sub-Center, Huang-Huai-Hai Key Laboratory of Biology and Genetic Improvement of Soybean, Ministry of Agriculture and Rural Affairs, Institute of Cereal and Oil Crops, Hebei Academy of Agricultural and Forestry Sciences, Shijiazhuang 050035, China; dragonyan1979@163.com; 6 Xuzhou Institute of Agricultural Sciences of Xu-Huai Region of Jiangsu, Xuzhou 221131, China

**Keywords:** soybean mosaic virus, RNAi, resistance, agronomic traits

## Abstract

The soybean mosaic virus (SMV), a significant viral pathogen impacting soybean cultivation, leads to substantial yield losses and diminishes seed quality. In a prior study, we developed a targeted silencing vector using RNA interference (RNAi) technology targeting the *CP* gene, which codes for the viral coat proteins in the SMV genome. This vector was delivered into soybean plants through *Agrobacterium*-mediated transformation. In our current research, we utilized ongoing molecular characterization and resistance screening to identify four genetically pure lines that display moderate to high resistance to SMV. Additionally, the transgenic plants exhibited resistance to three other potyviruses: the bean common mosaic virus, the recombinant soybean mosaic virus, and the watermelon mosaic virus. Greenhouse and field trials conducted with these lines demonstrated that RNAi-mediated silencing of the *CP* gene significantly enhanced disease resistance. It is noteworthy that, in comparison to the receptor plants, the transgenic plants exhibited no significant differences in maturity, plant height, branching number, node number, pod number, or 100-seed weight. These results offer valuable genetic resources and theoretical support for molecular breeding strategies aimed at combating SMV in soybeans, as well as for RNAi-based methods to control plant viral infections.

## 1. Introduction

Soybean mosaic virus (SMV) disease is a significant biotic stress affecting the global soybean industry. The disease typically results from a complex of viruses and has a severe impact [1]. The primary pathogen, SMV, is a member of the *Potyviridae* family and comprises a single-stranded RNA of the positive sense [2]. This virus spreads primarily through non-persistent aphid transmission and seed-borne vectors [3,4]. In major soybean-producing regions, SMV causes an average annual yield loss of 15–30%, with severe localized outbreaks exceeding 50% in some years, making it a critical constraint on stable and high-yield production [5,6]. Due to the lack of effective chemical control agents, traditional management heavily relies on breeding disease-resistant varieties. However, SMV exhibits complex strain differentiation and high mutation rates [7,8], posing potential risks that could allow new variants to overcome existing resistance.

RNA interference (RNAi) technology, which induces the degradation of target gene mRNA through a mechanism involving specific double-stranded RNA [9], has opened new avenues for plant virus-resistant breeding. In controlling tomato yellow leaf curl virus, constructing RNAi vectors targeting multiple viral genes increased disease resistance in transgenic plants by 80% [10]. For rice stripe virus resistance improvement, interference vectors targeting viral coat protein and movement protein genes enabled transgenic plants to exhibit full life-cycle immunity against the virus [11]. These findings demonstrate that RNAi technology can achieve cross-species and multi-target viral resistance enhancement with stable and long-lasting protective effects.

Recent advancements in RNAi technology for SMV research have achieved new breakthroughs in targeted interference of viral proteins and host susceptibility genes. The HC-Pro protein, serving as a viral gene-silencing suppressor [12], has become a key target for RNAi applications. Transgenic soybean lines developed with RNAi vectors targeting *HC-Pro* gene demonstrated significantly enhanced disease resistance against multiple SMV strains [13,14]. Notable advancements have also been realized in bolstering resistance to P3 protein interference; by employing RNAi to target the *P3* gene, the virus accumulation in transgenic soybeans was diminished by 72% [15]. Additionally, RNAi studies on the host susceptibility gene *GmVma12* revealed that transgenic soybeans exhibited stronger resistance to the SMV-SC15 strain, with markedly reduced seed mottling symptoms [16].

The coat protein (CP) of SMV serves as the primary structural component of viral particles. It plays crucial roles in viral particle assembly, stabilization, and mediating viral movement between host cells, while also acting as a key factor in viral–host interactions [17,18]. Sequence variations in the *CP* gene directly influence viral pathogenicity and strain differentiation, making it a critical target for antiviral research. In previous research, we constructed a vector that interfered with *CP* and conducted genetic transformation. In the present study, viruses were inoculated into transgenic soybean plants. The findings indicated that the transgenic plants exhibited moderate to high resistance against the prevalent SMV strains, resulting in a significant reduction in the accumulation of viruses within the plants and the appearance of symptoms. This provides an efficient technical solution for molecular breeding of virus-resistant soybeans.

## 2. Results

### 2.1. Generation of Transgenic Soybean Plants Containing the SMV CP Hairpin

**Figure 1:**
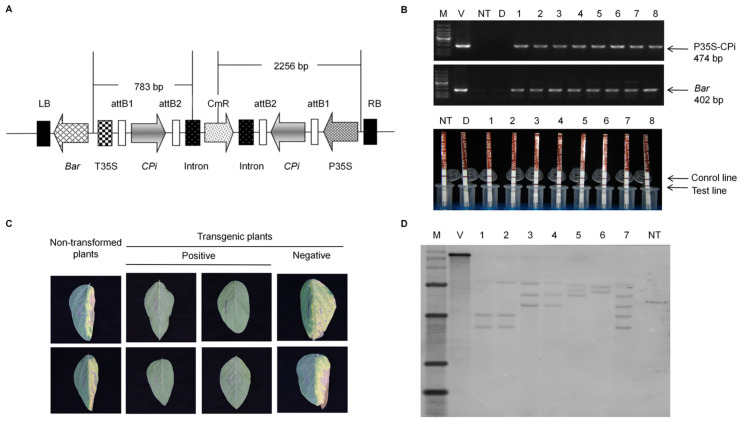
Presentation of RNAi vector and verification of transgenic plants. (**A**) Schematic representation of the T-DNA region of the recombinant pB7GWIWG2(II)-CPi plasmid used for soybean transformation [19]. The construct contains the *bar* gene conferring resistance to phosphoramphenol (PPT) and two 264-bp *CP* RNAi (CPi) fragments used to construct the hairpin structure. (**B**) PCR verification was carried out to detect the 474-bp CPi fragment and the 402-bp *bar* gene fragment in the genomic DNA of PPT-resistant plants. M marker D5000, V vector positive control, NT non-transformed plant negative control, D ddH_2_O blank control, 1–8 positive transgenic plants. LibertyLink^®^ strip detection was performed to analyze bar expression at the translational level. NT non-transformed plant negative control, D ddH_2_O blank control, 1–8 positive transgenic plants. (**C**) A leaf-painting assay was used to test for PPT-resistance in the putative transformants. Half of the leaf was painted with 200 mg/L PPT and the other half was marked with a black line, as the no-treatment control. (**D**) Southern blot analysis of transgenic T_1_ soybean plants. The genomic DNA of plants to be tested were digested with EcoRI and hybridized against a DIG-labeled CPi probe. M DNA molecular marker, V pB7GWIWG2(II)-CPi vector positive control, 1–7 positive transgenic plants, NT non-transformed plant negative control.

In previous studies, a 264-bp fragment (8732–8995 nt) was selected as the target sequence for constructing an RNAi expression vector containing its reverse repeat fragments, based on the conserved sequence of the SMV *CP* gene identified in GENBANK (Figure 1A) [19]. The SMV-CP RNAi (CPi) fragment was introduced into the soybean cultivar Huachun 6 using *Agrobacterium*-mediated transformation. A total of 2963 explants were transformed, and following differentiation, screening, and regeneration, 89 herbicide-resistant regenerated plants were obtained.

To obtain stable transgenic lines, continuous screening and purification of T_0_–T_3_ generation transgenic plants were conducted using PCR detection, LibertyLink^®^ strip analysis, and herbicide resistance testing. The PCR results indicated that transgenic plants exhibited the expected amplification for the CPi fragment (474 bp) and the *bar* gene (402 bp), corresponding to the predicted sizes. In contrast, no such bands were detected in the non-transformed plants (Figure 1B). The LibertyLink^®^ strip test results indicated that the transgenic plants were able to detect both the control and test lines. In contrast, only the control line was detected in the non-transformed plant (Figure 1B), confirming the presence of the bar protein in these plants, rather than its absence in the transgenic ones. The herbicide tolerance assay results demonstrated that the leaves of transgenic plants exhibited a distinct herbicide-resistant phenotype, following treatment with 200 mg/L PPT. In contrast, non-transformed plants exhibited significant yellowing and curling after herbicide application (Figure 1C). These results indicate that the RNAi-CP vector has been successfully transformed and is normally expressed in the plant.

Southern blot analysis was conducted to confirm that the *CP* hairpin was successfully integrated into the genome of T_1_ soybean plants. Seven PCR-positive transgenic soybean plants were selected for Southern blot hybridization, using the CPi sequence as the probe. The results indicated that all seven transgenic plants exhibited hybridization signals, whereas no bands were observed in the non-transformed plants (Figure 1D), signifying successful integration of the foreign fragment into the soybean genome. Additionally, the transgenic plants displayed two to four copies of T-DNA insertions.

### 2.2. The Transgenic Plants Exhibiting Resistance to Herbicides Also Demonstrated Significant Resistance to SMV-SC3

**Figure 2:**
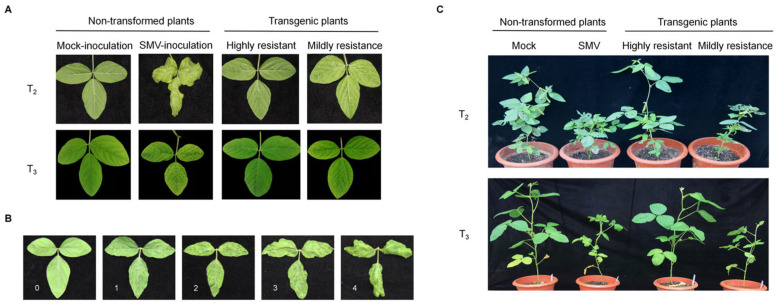
Responses of non-transformed and transgenic plants after inoculation with SMV-SC3 and disease rating classification. (**A**) The T_2_ and T_3_ generation plants, along with non-transformed plants, were inoculated with SMV-SC3. Symptoms of SMV infection were observed on the V_4_ leaves of both non-transformed and transgenic plants. Mock-inoculated non-transformed plants were used as the blank controls. (**B**) The disease rating was classified at five (0–4) grades, according to the infection severity in the leaves of the SC3-infected non-transformed plants. (**C**) Responses of the non-transformed and T_2_ and T_3_ plants 8 weeks after mechanical inoculation with SMV-SC3. Mock-inoculated NT plants were used as the blank controls.

To determine whether transgenic plants exhibit significantly different resistance to SMV compared to non-transformed plants, we conducted a resistance evaluation experiment. The visual responses of the fully expanded first (V_1_), second (V_2_), third (V_3_), and fourth (V_4_) trifoliate leaves of T_2_ and T_3_ transgenic plants and non-transformed plants inoculated with SMV-SC3 were observed. The results were as follows: 14 T_2_ generation plants and 32 T_3_ generation plants exhibited no symptoms of SMV infection and were classified as highly resistant (HR); 3 T_2_ generation plants showed mild early-stage symptoms that later resolved, classified as delayed resistance (DR); 44 T_2_ generation plants and 83 T_3_ generation plants displayed delayed mosaic or mild mosaic symptoms, classified as mildly resistant (MR); and 23 T_2_ generation plants and 2 T_3_ generation plants showed mosaic and curling symptoms throughout the four growth stages, classified as susceptible (S). The 90 non-transformed control plants exhibited susceptibility at all four stages (Table 1). Then, the symptoms of both non-transformed and transgenic plants inoculated with SMV-SC3 at the V_4_ stage were observed. Figure 2A shows that non-transformed plants displayed mosaic and curling symptoms in their V_4_ leaves. In contrast, the highly resistant T_2_ and T_3_ generation transgenic plants exhibited no mosaic symptoms and were indistinguishable from the mock-inoculated control plants. The mildly resistant T_2_ and T_3_ generation transgenic plants displayed relatively mild mosaic and curling symptoms.

Additionally, we conducted follow-up observations on the subsequent symptoms of the infected plants and performed virus content tests. The severity of mosaic disease in the top trifoliate leaves was evaluated at 8 weeks post-inoculation, as depicted in Figure 2B. Grade 0 indicates an absence of symptoms; Grade 1 signifies mild mosaic symptoms; Grade 2 denotes noticeable mosaic symptoms; Grade 3 features mosaic symptoms along with leaf curling; and Grade 4 indicates severe mosaic symptoms and leaf curling. The results showed that 84 T_2_ generation plants had an average disease severity of Grade 1, whereas 117 T_3_ generation plants exhibited no symptoms (Grade 0). In comparison, non-transformed plants averaged around Grade 4 in severity (Table 2 and Figure 2C). The accumulation of SMV in transgenic plants inoculated with SMV-SC3 and non-transformed plants inoculated with SMV-SC3 was detected using DAS-ELISA. The results indicated that the viral accumulation in T_2_ and T_3_ plants was less than double that of the negative control (non-transformed plants inoculated without SMV), whereas the viral accumulation in non-transformed plants was 10.6 times that of the negative control (Table 3). The results demonstrated that the transgenic positive plants exhibited resistance to herbicides and a high level of disease resistance to SMV-SC3.

### 2.3. The Transgenic Plants Exhibited Robust Resistance to Numerous Strains of SMV

**Figure 3:**
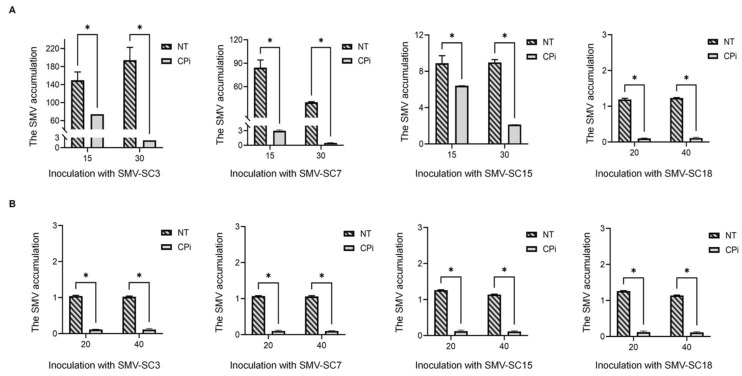
The levels of viral accumulation at different time points in T_3_ (CPi) plants and non-transformed (NT) plants were measured using qRT-PCR and DAS-ELISA. (**A**) The SMV accumulation at 15 and 30 days post-inoculation (dpi) in CPi and NT plants was quantified through qRT-PCR analysis targeting the *CP* gene of the SMV. Y axes indicate the SMV transcript levels of the CPi and NT plants infected with four SMV strains. X axes indicate the time points of virus content detection in CPi plants and NT plants. Data are expressed as the mean of three biological replicates with error bars indicating the standard deviation (SD). Asterisks represent significant differences between non-transformed and transgenic plants at the level of 0.05. (**B**) The SMV accumulation of CPi and NT at 20 and 40 dpi was detected using DAS-ELISA. Y axes indicate the SMV accumulation of the non-transformed and transgenic plants infected with four SMV strains. X axes indicate the time points of virus content detection in CPi plants and NT plants. Data are expressed as the mean of three biological replicates with error bars indicating the SD. Asterisks represent significant differences between non-transformed and transgenic plants at the level of 0.05.

Under dual testing conditions involving herbicides and SMV-SC3, four transgenic lines exhibiting high resistance to SMV-SC3 were identified. T_3_ generation plants from these lines were inoculated with other SMV strains (SMV-SC7, SMV-SC15, and SMV-SC18) to evaluate their resistance responses. The results indicated that at 15 and 30 dpi, the transgenic plants displayed significantly lower levels of SMV *CP* gene transcription compared to non-transformed plants. In particular, following inoculation with SMV-SC7 and SMV-SC18, the transgenic plants showed less than 0.1% of the *CP* gene transcription levels observed in non-transformed plants. Over time, by day 30 post-inoculation, the SMV *CP* gene transcription levels in the transgenic plants gradually decreased, whereas the non-transformed plants maintained relatively high levels (Figure 3A). Subsequently, the levels of viral accumulation in both transgenic and non-transformed plants were measured at 20 and 40 dpi. The results indicated that non-transformed plants sustained high viral accumulation at 20 and 40 dpi after inoculation with various strains of SMV, reaching levels 9.5 to 11.9 times higher than those observed in transgenic plants (Figure 3B). These findings suggest that transgenic plants demonstrate robust resistance to multiple strains of SMV.

### 2.4. The Transgenic Plants Infected with Potyviruses Maintained the Original Agronomic Traits and Yield of the Recipient Plants

**Figure 4:**
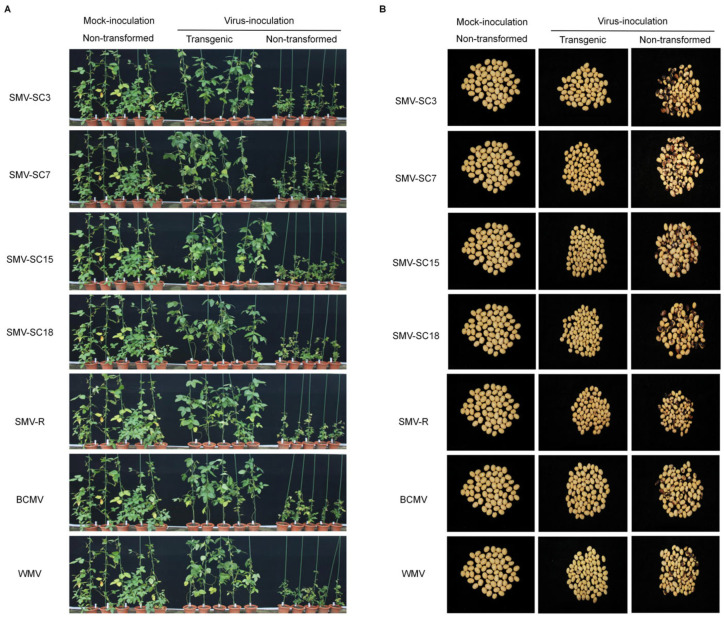
Phenotypes and seeds of transgenic and non-transformed plants inoculated with four SMV strains, BCMV, SMV-R, and WMV, in the greenhouse. (**A**) Phenotypes of transgenic and non-transformed plants inoculated with four SMV strains, BCMV, SMV-R, and WMV, in the greenhouse. Mock-inoculated non-transformed plants were used as the blank controls. (**B**) T_4_ seeds harvested from virus-inoculated transgenic plants and non-transformed plants.

**Figure 5:**
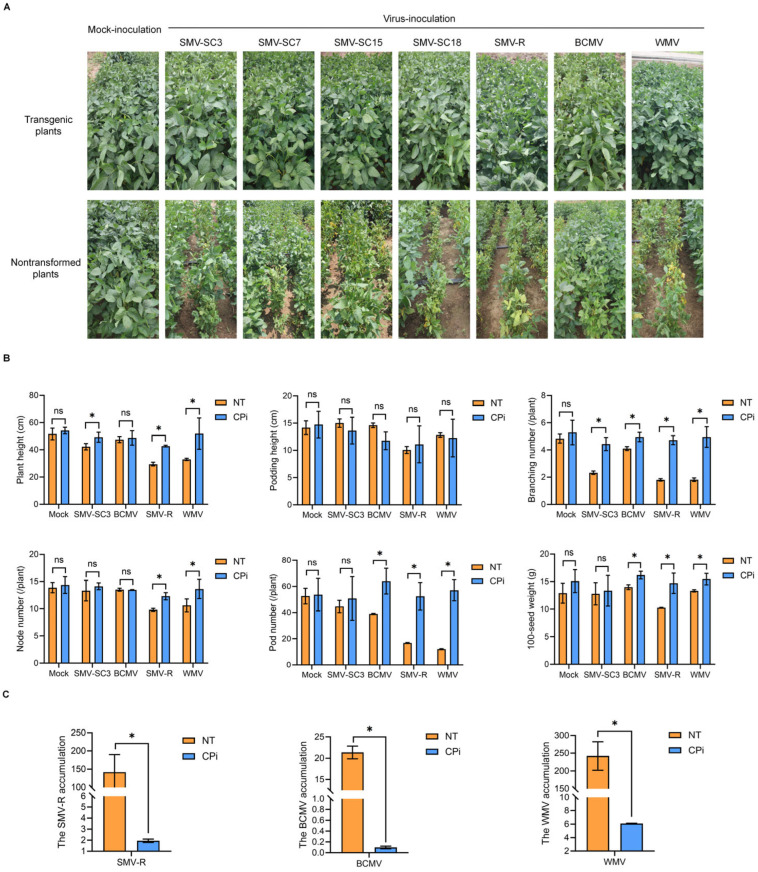
Phenotypes of transgenic and non-transformed plants inoculated with four SMV strains, BCMV, SMV-R, and WMV, under field conditions. (**A**) Phenotypes of transgenic and non-transformed plants inoculated with four SMV strains, BCMV, SMV-R, and WMV, in the field. Mock-inoculated non-transformed plants served as the blank controls. (**B**) SMV-SC3, BCMV, SMV-R, and WMV were inoculated into transgenic (CPi) plants and non-transformed (NT) plants, respectively, and their key agronomic traits were investigated post-harvest. Y axes indicate the data of key agronomic traits. X axes indicate the categories of agronomic traits. Data are expressed as the means of at least nine tested plants, with error bars indicating the SD. The asterisk indicates significant differences at the 0.05 level between non-transformed and transgenic plants, while ‘ns’ indicates no significant differences among treatments. (**C**) The viral accumulation at 30 dpi in transgenic (CPi) and non-transformed (NT) plants inoculated with SMV-R, BCMV, or WMV was quantified via qRT-PCR. Y axes indicate the viral accumulation in the CPi and NT plants infected with SMV-R, BCMV, or SMV. Y axes indicate the virus content in CPi plants and NT plants. Data are presented as the mean of three biological replicates, with error bars denoting the SD. Asterisks signify significant differences between non-transformed and transgenic plants at the 0.05 level.

Transgenic T_4_ generation plants and non-transformed plants were planted both in greenhouses and fields, and inoculated with multiple potyviruses—four SMV strains, BCMV, SMV-R, and WMV—to investigate differences in agronomic traits between the two groups. These traits included maturity period, plant height, podding height, branching number, node number, pod number, and 100-seed weight. The results indicated that non-transformed plants in both greenhouse and field settings exhibited leaf curling and stunted growth after inoculation with different viruses, whereas the transgenic plants did not display stunting symptoms similar to those of their non-transformed counterparts (Figure 4A and Figure 5A). A morphological analysis of the harvested seeds indicated that non-transformed seeds inoculated with viruses primarily developed brown seed-coat mottling, whereas transgenic seeds inoculated with these viruses rarely showed such symptoms (Figure 4B).

A comparative agronomic analysis was conducted between non-transformed plants and transgenic plants subjected to mock treatment. The results demonstrated that no significant differences were observed between the transgenic and non-transformed plants in key traits such as plant height, podding height, branching number, nod number, pod number, 100-seed weight, and duration of maturity (Figure 5B, Table S2). However, it is noteworthy that non-transformed plants inoculated with different viruses exhibited reduced plant height, branching number, pod number, and 100-seed weight compared to transgenic plants inoculated with the same virus (Figure 5B).

Following inoculation with four viral strains (SMV-SC3, BCMV, SMV-R, and WMV), the agronomic performance of non-transformed plants was compared with that of transgenic lines, to evaluate the differences in viral resistance. The results showed that non-transformed plants suffered significant reductions in multiple agronomic traits relative to transgenic plants, with the damage severity varying substantially among virus treatments. Specifically, after SMV-SC3 inoculation, non-transformed plants exhibited a 14.24% decrease in plant height and a 47.40% reduction in branching number. Under BCMV infection, the branching number, pod number and 100-seed weight of non-transformed plants declined by 17.17%, 39.11% and 13.54%, respectively. Notably, SMV-R caused the most severe damage to non-transformed plants, leading to reductions of 30.96% in plant height, 61.86% in branching number, 20.33% in nod number, 67.73% in pod number, and 30.27% in 100-seed weight. In the WMV treatment group, non-transformed plants showed the largest declines in plant height (36.69%) and pod number (78.74%), accompanied by decreases of 63.43% in branching number, 22.08% in nod number, and 11.78% in 100-seed weight. Collectively, these findings indicated that transgenic soybean plants had stronger resistance to the tested viral strains than non-transformed plants.

Additionally, both non-transformed and transgenic plants exhibited no significant differences in podding height, regardless of their status of inoculation with different viruses (Table S2). The findings suggest that transgenic plants do not display significant differences in key agronomic traits compared to non-transformed plants, and their inherent resistance to potyviruses contributes to more effectively stabilizing soybean yield and quality.

## 3. Discussion

Current research has identified major resistance genes against SMV located on chromosomes 2, 13, and 14 [20,21,22]. The resistance gene on chromosome 2 encodes an RNase that interacts with SMV’s P3 protein, degrading double-stranded RNA during viral replication to trigger resistance responses [23]. However, this resistance has been overcome by emerging variants [24]. Resistance genes on chromosomes 13 and 14 are confirmed as nucleotide-binding domain and leucine-rich repeat genes [25,26], which mediate resistance through interactions with SMV’s HC-Pro and CI proteins, respectively [27,28]. Nevertheless, the large genomic size of these two resistance genes makes it challenging to develop resistant varieties through conventional genetic engineering techniques [29]. Compared to the introduction of resistance genes into host plants, RNAi technology has demonstrated numerous significant advantages in the field of resistance breeding research. Firstly, RNAi technology requires relatively simple operations, eliminating the need for complex gene cloning and transformation procedures. By simply designing and introducing appropriate interference sequences, research cycles can be significantly shortened and costs reduced [30]. Secondly, RNAi technology has high specificity, allowing for precise targeting of specific viral genes, which effectively inhibits viral replication and transmission without adversely affecting other host plant genes. In contrast, introducing resistance genes may trigger unforeseen genetic interactions that could disrupt the normal growth and development of the host plant [31]. Furthermore, RNAi technology offers broad-spectrum resistance coverage. A single interference vector can be engineered to target multiple viral genes or different virus genera simultaneously, achieving multi-resistance or broad-spectrum protection [32]. These advantages ensure that RNAi technology continues to play a significant role in the field of resistance research and breeding applications.

The *CP* gene of the soybean mosaic virus is crucial for its replication, assembly, and movement between cells. To enhance plant defenses against pathogens, researchers have employed two primary strategies—gene silencing and gene overexpression—to investigate the operational mechanisms of the *CP*. The technique of gene silencing adeptly curtails *CP* expression via targeted RNAi pathways, leading to a diminished production of viral coat proteins and a marked decrease in the virus’s reproductive efficiency within the plant [33,34]. In contrast, the strategy of gene overexpression increases *CP* transcription in plant cells through genetic modification, thereby enhancing the plants’ ability to resist viral infections [35]. Findings have revealed that silencing the *CP* in plants significantly increased the resistance of transgenic soybeans to the SMV, effectively halting viral replication and transmission. Similarly, the overexpression of *CP* also endowed transgenic plants with enhanced viral resistance, presumably due to the surplus *CP* disrupting the viral lifecycle or provoking plant immune responses, thus restraining viral growth and dissemination [36]. In summary, these findings offer a robust theoretical framework and technical support for the development of novel virus-resistant soybean cultivars.

This study successfully developed an efficient RNAi vector targeting the SMV *CP* gene and obtained genetically stable resistant lines through *Agrobacterium*-mediated soybean transformation. When inoculated with multiple SMV strains, these resistant lines demonstrated enhanced SMV resistance compared to non-transformed plants. Previous studies have also demonstrated that interfering with the *CP* gene in SMV can produce similar resistance-enhancement effects [33,34], further confirming that RNAi-CP is an effective strategy for resistance improvement and breeding. Compared with similar studies, our work innovatively maintains key agronomic traits in resistant lines, while effectively preserving soybean yield and seed quality compared to susceptible plants.

Through sequence alignment (Figure S1), four SMV strains, SMV-R, BCMV, and WMV, were found to exhibit high sequence homology with one another. Among these strains, SMV-SC3 shared 100% homology with the *CP* interference fragment, whereas BCMV showed the lowest homology, at 75%; the remaining viral strains displayed homology levels ranging from 86% to 97%. This high degree of sequence similarity suggests that the *CP* genes of these viruses are susceptible to RNAi-mediated silencing upon invading the transgenic plants, which consequently confers broad-spectrum viral resistance to the transgenic plants.

Notably, non-transformed plants inoculated with BCMV, SMV-R, or WMV all exhibited typical dwarfing phenotypes, but the extent of agronomic trait damage varied distinctly among the viral treatments. Specifically, non-transformed plants infected with SMV-R and WMV suffered severe and comprehensive damage to multiple agronomic traits, including plant height, branching number, node number, pod number, and 100-seed weight. In contrast, BCMV infection only affected the branching number, pod number, and 100-seed weight of non-transformed plants, with no significant alterations detected in plant height and node number.

Consistent with the phenotypic observations, qRT-PCR analysis revealed that the viral mRNA accumulation levels in non-transformed plants were significantly higher than those in transgenic plants across all three viral treatments (Figure 5C). Interestingly, the viral mRNA abundance in BCMV-infected non-transformed plants was notably lower than that in plants inoculated with SMV-R or WMV. Based on these findings, we hypothesize that BCMV exhibits relatively lower virulence in the soybean cultivar Huachun 6 compared with SMV-R and WMV, which accounts for the less severe alterations in agronomic traits observed in BCMV-infected plants.

## 4. Materials and Methods

### 4.1. Materials

The viruses used in this study were SMV-SC3, SMV-SC7, SMV-SC15, SMV-SC18, recombinant soybean mosaic virus (SMV-R), bean common mosaic virus (BCMV), and watermelon mosaic virus (WMV), as well as *Agrobacterium* EHA105 and interference expression vector pB7GWIWG2 (II), which were provided by the National Soybean Improvement Center of Nanjing Agricultural University (Nanjing, China). The transgenic recipient Huachun 6 was kindly provided by Professor Hai Nian from South China Agricultural University (Guangzhou, China). The *Escherichia coli* DH5α used for vector construction and the intermediate vector pDONR221 were purchased from TianGen Biochemical Technology Co., Ltd. (Beijing, China) and Invitrogen Corporation, Inc. (Carlsbad, CA, USA), respectively.

### 4.2. Identification of Transgenic Plants

In our initial studies, we effectively assembled an interference vector, pB7GWIWG2(II)-CPi, which includes the *CP* gene fragment. The recombinant vector pB7GWIWG2(II)-CPi contains a plant-selection marker, namely the herbicide resistance gene *bar*, which confers resistance to phosphinothricin (PPT) (Figure 1A). By employing a cotyledonary node–*Agrobacterium*-mediated soybean transformation system, we successfully generated T_0_ transgenic soybean plants. For comprehensive details on vector construction and tissue culture methods, please consult the research by Gao et al. [19].

The T_1_–T_3_ generation of transgenic plants was cultivated in the greenhouse at Pailou, Nanjing Agricultural University. At the first trifoliate leaf stage, the transgenic plants were identified using the leaf-painting method, the LibertyLink^®^ strip detection method, and the PCR detection method. Pure lines were then selected for further study.

The leaf-painting method involved diluting the PPT solution to a concentration of 200 mg/L. One half of a leaf was marked using a marker pen, with the main leaf vein serving as the boundary, and the other half was brushed with the diluted solution. After one week of cultivation, the reaction of the herbicide on the leaves was observed.

LibertyLink^®^ strip detection was conducted using a QuickStix^TM^ Kit (EnviroLogix, Inc. (Portland, ME, USA)) to verify the expression of the *bar* gene. The process includes the following steps: place 100 mg of fresh leaf tissue into a 1.5 mL centrifuge tube. Using a grinding rod, rotate the tube to extract the juice, then add 0.5 mL of extraction buffer for dilution and mixing. Insert the test strip into the centrifuge tube, following the arrow direction. After a 5 min incubation period, observe whether the detection line develops coloration.

The PCR detection protocol involves extracting DNA from fresh leaves at 100 mg/L using an efficient plant genomic DNA extraction kit (DP350, TianGen Biochemical Technology Co., Ltd. (Beijing, China)). The insertion fragments of CPi and *bar* were amplified using Premix Taq (R004A, TaKaRa Bio, Inc. (Beijing, China)), with detection primers 35S-P/attB2-CPi-R and bar-F/bar-R synthesized by General Biotech (Anhui) Co., Ltd. (Anhui, China). The reaction system consisted of 25 μL Premix Taq, 1 μL template (<500 ng), 1 μL each of upstream and downstream primers, and 22 μL sterilized water. The reaction was amplified on a T100^TM^ gradient PCR instrument (Bio-Rad Laboratories, Inc. (Hercules, CA, USA)) with the following program: 98 °C pre-denaturation 10 s, 55 °C denaturation 30 s, 72 °C extension 1 min, for 30 cycles. The products were analyzed by 1% agarose gel electrophoresis, and the DNA molecular markers used were purchased from Sangon Biotech (Shanghai) Co., Ltd. (Shanghai, China).

### 4.3. Southern Blot Hybridization Analysis

Genomic DNA from T_1_ generation transgenic plants was extracted using an efficient plant genome DNA extraction kit. Approximately 30 μg of DNA samples were digested with EcoRI enzyme (Thermo Fisher Scientific, Inc. (Waltham, MA, USA)), which specifically cleaves the T-DNA region. The digested DNA was electrophoretically separated on a 0.8% agarose gel and transferred to Hybond-N+ nylon membrane (Amersham, (Buckinghamshire, UK)). A 264-bp fragment containing the *CP* gene coding region of pB7GWIWG2(II)-CPi plasmid was amplified using attB1-CPi-F/attB2-CPi-R primers. Primer sequence information is shown in Table S1. The product was subsequently labeled with digoxin (DIG) for high-intensity detection (Hoffmann-La Roche, Inc. (Indianapolis, IN, USA)), and the labeled product was used as the probe. Subsequent hybridization, film washing, and signal detection were performed according to the instructions provided in the DIG High Prime DNA Labeling and Detection Kit II (Hoffmann-La Roche, Inc. (Indianapolis, IN, USA)).

### 4.4. SMV Inoculation and Resistance Assessment

T_2_–T_3_ generation plants and non-transformed plants were grown in greenhouses. After the young leaves unfolded, SMV-SC3 was inoculated using the methodology described by Gao et al. (2015) [13]. The responses of the T_2_ and T_3_ plants were visually observed during the V_1_–V_4_ stages. These responses were classified into four categories: HR-type (highly resistant to SMV, with no viral symptoms observed during the study period); DR-type (delayed resistance manifestation, with early viral symptoms that later disappeared); MR-type (mildly resistant, with delayed viral symptoms or mild symptoms compared to susceptible controls); and S-type (highly susceptible, with viral symptoms consistent with controls across all four stages). Additionally, the severity ratings for SMV were assessed on the top three fully developed leaves at 8 weeks post-inoculation (the topmost, second, and third layers), with the average leaf ratings indicating the overall plant infection levels. The disease severity was categorized into five grades: Grade 0 (asymptomatic); Grade 1 (mild mosaic symptoms); Grade 2 (mosaic symptoms); Grade 3 (mosaic symptoms with leaf curling); and Grade 4 (mosaic symptoms with severe leaf curling).

### 4.5. Quantitative Real-Time PCR (qRT-PCR) Analysis

Four SMV strains (SMV-SC3, SMV-SC7, SMV-SC15, and SMV-SC18) were inoculated into T_3_ generation transgenic plants and non-transformed plants. Leaves were collected at 15 and 30 days post-inoculation (dpi), and the viral accumulation in the plants was detected using qRT-PCR. Samples were transferred to centrifuge tubes, ground into powder with liquid nitrogen, and processed for RNA extraction and cDNA synthesis. Amplification was performed using the primers CP-testF/CP-testR and Tubulin-F/Tubulin-R (as the internal reference gene), with the CFX96 Touch real-time PCR detection system (Bio-Rad Laboratories, Inc. (Hercules, CA, USA)). In addition, leaves were collected from both non-transformed and transgenic plants at 30 dpi with SMV-R, BCMV, and WMV, respectively. The viral accumulation levels of these three viruses in the leaves were then detected using the primers CP-testF/CP-testR, BCMV-CP-testF/BCMV-CP-testR, and WMV-CP-testF/WMV-CP-testR, which correspondes to each virus. The data were analyzed employing the 2^−ΔΔCT^ method.

### 4.6. Serological Determination

Four SMV strains (SMV-SC3, SMV-SC7, SMV-SC15, and SMV-SC18) were inoculated into the T_3_ generation of transgenic plants and non-transformed plants. Leaves were collected at 20 and 40 dpi, and the viral accumulation in the plants was detected using a double-antibody sandwich enzyme-linked immunosorbent assay (DAS-ELISA) kit (V094, NanoDian, Inc. (Los Angeles, CA, USA)). The collected leaves were placed in sampling tubes, ground into a powder, and analyzed for viral content using the Infinite^®^ 200 PRO (Tecan Group Ltd. (Männedorf, ZH, Switzerland)) with an OD405 nm reading. The values of transgenic and non-transformed plants were converted to multiples of the negative control. Samples with relative values exceeding twice the negative control were classified as positive.

### 4.7. Agronomic Traits of Transgenic Plants Under Greenhouse and Field Conditions

Transgenic T_4_ plants and non-transformed plants were cultivated under greenhouse and field conditions, respectively. Four strains of soybean mosaic virus and three other potyviruses—bean common mosaic virus (BCMV), recombinant soybean mosaic virus (SMV-R), and watermelon mosaic virus (WMV)—were inoculated into the transgenic lines and non-transformed plants. Forty days post-inoculation, both transgenic and non-transformed plants were photographed simultaneously. During harvest, at least nine plants from each treatment group were randomly selected for agronomic trait evaluation. Measurements were taken for maturity period, plant height, podding height, branching number, node number, pod number, and 100-seed weight (Table S2). Seed samples from both inoculated and non-inoculated plants were also collected for seed-coat mottling analysis.

### 4.8. Statistical Analysis

The Jamovi software (Version 1.6) [37] was utilized to analyze the differences in resistance levels between transgenic soybean plants and control recipient varieties, using a two-tailed Student’s *t*-test. Multiple comparisons were conducted using the least significant difference method when examining the variance of agronomic traits across various treatment groups. Variations were deemed statistically significant if the *p*-value was less than 0.05.

## 5. Conclusions

This study successfully generated four genetically stable, high-resistance lines by employing RNAi-mediated specific silencing of the SMV *CP* gene. The primary conclusions are as follows: firstly, the constructed pB7GWIWG2(II)-CPi vector can efficiently induce post-transcriptional silencing of the *CP* gene in soybean cells, significantly inhibiting SMV replication. Secondly, the screened resistant lines maintained normal growth and development under viral stress, with their critical agronomic traits consistent with those of the recipient. These findings not only provide new genetic resources and breeding materials for molecular breeding of soybean resistance to SMV, but also offer a replicable technical system for utilizing RNAi technology to control plant viral diseases.

## Figures and Tables

**Table 1:** Classification of the response types of transgenic plants to SMV strain SC3 at the V_1_–V_4_ stage.

Plant Types	Numbers of Plants Evaluated	HR ^a^	DR ^b^	MR ^c^	S ^d^
CPi-T_2_	84	16.7% (14)	3.6% (3)	52.4% (44)	27.4% (23)
CPi-T_3_	117	27.4% (32)	0 (0)	70.9% (83)	1.7% (2)
NT	90	0 (0)	0 (0)	0 (0)	100.0% (90)

CPi-T_2_ T_2_ generation transgenic plants; CPi-T_3_ T_3_ generation transgenic plants; NT non-transformed plants. ^a^ Highly resistant to SMV, indicating no visible symptoms (i.e., mosaic, chlorosis, curl and necrosis) appeared on soybean leaves at all the four stages. ^b^ Delayed resistance to SMV, indicating symptoms appeared at an early stage and disappeared at later stages. ^c^ Mildly resistance to SMV, indicating delayed appearance of symptoms or symptoms lighter than those of the susceptible controls. ^d^ Susceptible to SMV, indicating plants were as symptomatic as the susceptible controls at all four stages.

**Table 2:** Calculation of the average disease rating of plants inoculated with SMV-SC3.

Plant Types	Numbers of Plants Evaluated ^a^	Average Disease Rating ^b^
CPi-T_2_	84	0.90 (±0.30)
NT ^c^	50	3.62 (±0.26)
CPi-T_3_	117	0.16 (±0.42)
NT ^d^	40	3.87 (±0.35)

CPi-T_2_ T_2_ generation transgenic plants; CPi-T_3_ T_3_ generation transgenic plants; NT non-transformed plants. ^a^ Disease rating of each plant was calculated by averaging the disease ratings of the top three leaves. ^b^ Disease rating of each generation was calculated by averaging all the disease ratings of plants. ^c^ Non-transformed plants were used as controls for T_2_ transgenic plants for resistance assessment of the top three leaves. ^d^ Non-transformed plants were used as controls for T_3_ transgenic plants for resistance assessment of the top three leaves.

**Table 3:** DAS-ELISA analyses of T_2_ andT_3_ transgenic plants previously inoculated with SMV-SC3, based on the optical density value.

Plant Types	P (OD405 nm) ^a^	N (OD405 nm) ^b^	P/N
CPi-T_2_	0.11 (±0.00)	0.11 (±0.01)	1.04 (-)
CPi-T_3_	0.09 (±0.01)	0.10 (±0.01)	0.98 (-)
NT	1.05 (±0.01)	0.10 (±0.01)	10.61 (+)

CPi-T_2_ T_2_ generation transgenic plants; CPi-T_3_ T_3_ generation transgenic plants; NT non-transformed plants; + positive for SMV; - negative for SMV. ^a^ OD405 nm value of each plant was calculated by averaging the OD405 nm values of five SMV-inoculated plants randomly selected in the line. ^b^ OD405 nm value of each negative control was calculated by averaging the OD405 nm values of three mock-inoculated plants (negative samples).

## Data Availability

The original contributions presented in this study are included in the article. Further inquiries can be directed to the corresponding author.

## Supplementary Materials

The following supporting information can be downloaded at: https://www.mdpi.com/article/10.3390/plants15030430/s1, Table S1: The primers used in this study; Table S2: Agronomic performance of non-transformed and transgenic plants under field conditions; Figure S1: Sequence alignment of *CP* interference fragments among four SMV strains, SMV-R, BCMV, and WMV.

## Abbreviations

The following abbreviations are used in this manuscript:

BCMV	Bean common mosaic virus
CP	Coat protein
Dpi	Day post-inoculation
DAS-ELISA	Double-antibody sandwich enzyme-linked immunosorbent assay
RNAi	RNA interference
SMV	Soybean mosaic virus
SMV-R	Recombinant soybean mosaic virus
WMV	Watermelon mosaic virus
