# Supplementary figures and images for: Enhanced Soybean Immunity to the Soybean Mosaic Virus Through RNA Interference Targeting the CP Gene
Source: Plants (Basel). 2026 Jan 30;15(3):430. doi: 10.3390/plants15030430 (PMC12899861; doi:10.3390/plants15030430)

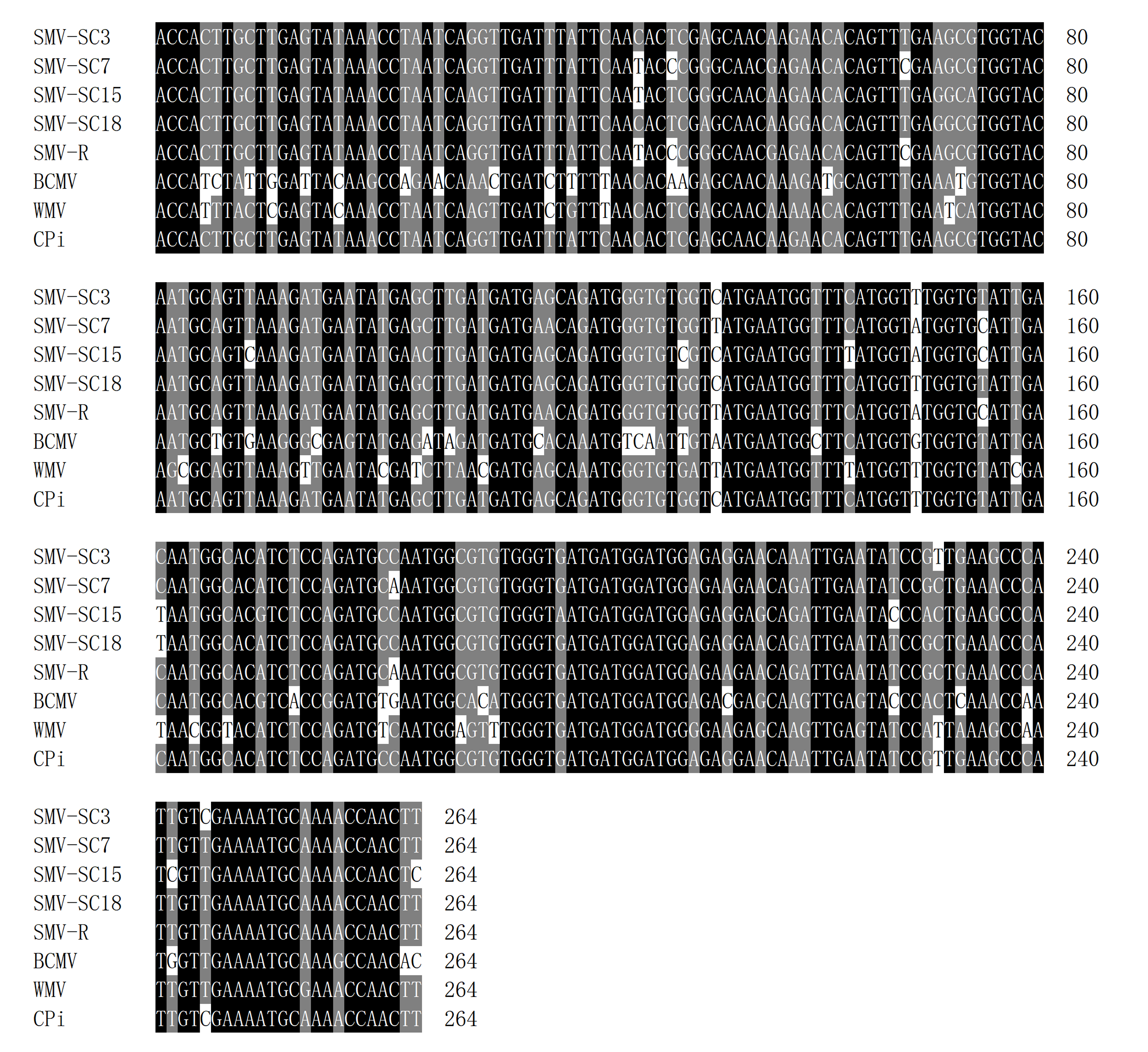

Supplement: Supplementary file 1 [file plants-15-00430-s001.zip › Figure S1.png]
